# Feeding butter with elevated content of trans-10, cis-12 conjugated linoleic acid to obese-prone rats impairs glucose and insulin tolerance

**DOI:** 10.1186/s12944-015-0122-2

**Published:** 2015-09-28

**Authors:** Melissa Hamilton, Loren E. Hopkins, Ousama AlZahal, Tara L. MacDonald, Daniel T. Cervone, David C. Wright, Brian W. McBride, David J. Dyck

**Affiliations:** Department of Human Health and Nutritional Sciences, University of Guelph, Guelph, Ontario N1G2W1 Canada; Department of Animal and Poultry Science, University of Guelph, Guelph, Ontario N1G2W1 Canada

**Keywords:** Conjugated linoleic acid, CLA (t10,c12), Glucose tolerance, Insulin tolerance, Insulin-stimulated glucose uptake, Female Zucker rats

## Abstract

**Background:**

We recently demonstrated that feeding a natural CLA_t10,c12_-enriched butter to lean female rats resulted in small, but significant increases in fasting glucose and insulin concentrations, and impaired insulin tolerance. Our goal was to extend these findings by utilizing the diabetes-prone female fatty Zucker rat. Rats were fed custom diets containing 45 % kcal of fat derived from control and CLA_t10,c12_-enriched butter for 8 weeks.

**Methods:**

CLA _t10,c12_-enriched butter was prepared from milk collected from cows fed a high fermentable carbohydrate diet to create subacute rumen acidosis (SARA); control (non-SARA) butter was collected from cows fed a low grain diet. Female fatty Zucker rats (10 weeks old) were randomly assigned to one of four diet treatments: i) low fat (10 % kcal), ii) 45 % kcal lard, iii) 45 % kcal SARA butter, or iv) 45 % kcal non-SARA butter. A low fat fed lean Zucker group was used as a control group. After 8 weeks, i) glucose and insulin tolerance tests, ii) insulin signaling in muscle, adipose and liver, and iii) metabolic caging measurements were performed.

**Results:**

Glucose and insulin tolerance were significantly impaired in all fatty Zucker groups, but to the greatest extent in the LARD and SARA conditions. Insulin signaling (AKT phosphorylation) was impaired in muscle, visceral (perigonadal) adipose tissue and liver in fatty Zucker rats, but was generally similar across dietary groups. Physical activity, oxygen consumption, food intake and weight gain were also similar amongst the various fatty Zucker groups.

**Conclusions:**

Increasing the consumption of a food naturally enriched with CLA_t10,c12_ significantly worsens glucose and insulin tolerance in a diabetes-prone rodent model. This outcome is not explained by changes in tissue insulin signaling, physical activity, energy expenditure, food intake or body mass.

## Background

Conjugated linoleic acid (CLA) refers to the geometric and positional isomers of linoleic acid (C18:2 n-6). The most abundant naturally occurring CLA isomers are CLA_c9,t11_ (76 %) and CLA_t10,c12_ (1 %) [1, 2], and are derived from food sources containing ruminant fat [3]. Early studies demonstrated that supplementation of CLA in the diets of mice caused massive losses of body fat, essentially creating a lipoatrophic condition [4–6]. Indeed, such findings fueled a subsequent interest in supplemental CLA as a potential tool to combat obesity. However, the weight loss effects of supplemental CLA in humans is much more modest [7–10]. Furthermore, an unexpected outcome of the earlier CLA studies was an increase in indices of insulin resistance, which was evident in mice [4, 5] and overweight humans [7–10], and generally attributed to the CLA_t10,c12_ isomer [11]. Conversely, there is evidence that the CLA_c9,t11_ isomer may confer insulin sensitizing effects [12].

The majority of studies to date have examined the effects of supplemental CLA, as opposed to examining the outcome of consuming food naturally enriched with CLA. More specifically, almost no studies have examined the metabolic consequences of consuming a food source in which the CLA isomer content has been naturally altered i.e. not merely adding CLA isomer in the chemical form to a food. To this end, we recently examined the impact of feeding a CLA_t10,c12_-enriched butter (60 % of total kcal) to lean, healthy female rats. Our findings indicated that feeding the CLA_t10,c12_-enriched butter resulted in small, but significant increases in fasting glucose and insulin concentrations, as well as impaired insulin tolerance [13]. However, there was no effect of the enriched butter on glucose or pyruvate tolerance, or on insulin-stimulated glucose uptake in muscle [13]. Shortly after our study was published, there was another report showing that feeding butter enriched in the CLA_c9,t11_ isomer to lean rats prevented the hyperinsulinemia induced by a high fat diet; interestingly, there was also no effect on oral glucose tolerance in this study [14]. Collectively, these two studies demonstrate a potential, albeit relatively small, effect of consuming natural food sources with altered CLA isomer content on indices of insulin sensitivity.

Given the relatively small effect of CLA_t10,c12_-enriched butter on indices of insulin resistance in lean, healthy rats in our previous study [13], we decided to next examine the impact of feeding this potentially “less healthy” butter to fatty female Zucker rats, which develop insulin resistance when placed on a high fat diet [15]. We speculated that the general lack of negative consequences to feeding SARA butter in our previous study was due to the choice of lean rats as a model, and that a rodent model with a predisposition towards diabetes would be more likely to show negative effects on glucose and insulin tolerance, and insulin signaling. As in our previous study [13], we fed dairy cows a high grain, low forage diet to induce a condition known as subacute rumenal acidosis (SARA), which is defined as a decrease in rumenal pH below 5.6 for 4–6 hrs during a 24-h period [16, 17]. This decrease in pH results in a significant increase in the production of the CLA_t10,c12_ isomer due to incomplete biohydrogenation of polyunsaturated fatty acids [18].

Overall, our goal was to extend our previous findings by investigating the impact of a CLA _t10,c12_-enriched butter on glucose and insulin tolerance, as well as insulin signaling in muscle (oxidative and glycolytic), adipose (subcutaneous and visceral) and liver tissues, in an obese, diabetes prone rat model. To this end, female fatty Zucker rats were fed custom diets containing high amounts of fat (45 % of total kcal) derived from SARA and non-SARA butter for 8 weeks. We hypothesized that, unlike lean insulin sensitive rats, fatty Zucker rats would demonstrate a clear worsening of both glucose and tolerance, as well as impaired insulin signaling in muscle, adipose and liver.

## Methods

### Ethics statement

All procedures were carried out in accordance with the recommendations of the Canadian Council of Animal Care, and were approved by the Animal Care Committee at the University of Guelph (Animal Utilization Protocols 09R046 and 1515). All surgeries on rodents were performed under sodium pentobarbital anesthesia and all efforts were made to prevent discomfort and suffering.

### Feeding of cows, milk collection and processing

Procedures for feeding cows and collecting milk were the same as reported in our previous study [13]. Six lactating Holstein cows housed at the Ponsonby Dairy Research Centre, University of Guelph, Guelph, Ontario were used in this study. The cows were randomly assigned to one of two dietary treatments for a total of 21 days. The two diets were: i) a high-forage/low-grain diet (*n* = 4 cows) designed to promote a stable and optimal rumenal pH, or ii) a SARA-inducing high-grain diet containing readily fermentable carbohydrates (*n* = 2 cows) intended to reduce rumen pH resulting in a shift in the biohydrogenation pathway leading to elevated levels of CLA_t10,c12_ in milk fat. Milk collection took place on days 13 through 21. Two to four cows provided sufficient milk to make the necessary quantities of butter to perform the current and previous study [13]. The milk from each cow was collected separately, placed on ice, and transferred to the University of Guelph Food Technology Centre where it was pooled by treatment and processed to make unsalted butter; the fatty acid profiles were analyzed and previously reported [19]. All animals were handled and cared for in accordance with the Canadian Council on Animal Care regulations, and as approved the Animal Care Committee at the University of Guelph.

### Experimental animal groups

Eight female lean Zucker rats and 36 female fatty Zucker rats from Charles River (Quebec, Canada) were used in this study. Rats were 10 weeks old upon arrival and were randomly assigned 2–3 rats per cage in a room with a 12-h light/dark cycle. Rats were allowed free access to water and a low fat (10 % kcal) diet for a 1-week acclimatization period. Subsequently, for an 8-week period, the lean Zucker rats were fed the low fat diet and the fatty Zucker rats were randomly assigned to one of four diet treatments: i) low fat (10 % kcal, LF), ii) 45 % kcal lard (LARD) as a positive control *i.e.* one in which we expected to see the development of glucose and insulin intolerance, iii) 45 % kcal SARA butter (SARA), or iv) 45 % kcal non-SARA butter (non-SARA). Animals were allowed ad libitum access to food and water. Food intake (daily) and body mass (weekly) were monitored regularly throughout the treatment period.

### Diet preparation

Two butter diets (non-SARA, SARA) were made in order to evaluate the physiological responses (glucose and insulin tolerance, insulin signaling) from consuming healthy and potentially unhealthy butters. A purified low fat diet (Research Diets D12450B, New Brunswick, New Jersey) containing 10 % kcal from fat (5.5 % soybean oil and 4.5 % lard) and a purified high fat diet (Research Diets D12451) containing 45 % kcal from fat (5.5 % soybean oil and 39.5 % lard) were used for this study. A premix (Research Diets D12451px) identical in composition to the high fat diet, excluding the fat content, was used to prepare the custom 45 % kcal non-SARA and SARA butter diets. To make each custom butter diet, 1.61 kg of each type of butter was mixed with 5.12 kg of premix. Diets were partitioned into small airtight containers and stored at −20 °C until use.

### Glucose tolerance test

After a 6 h fast, blood glucose was measured from the tail of each rat before receiving a bolus injection of glucose (2 g/kg) into the intraperitoneal cavity. Blood glucose concentrations were measured with glucose strips and a glucometer (Freestyle Lite, Abbott Diabetes Care Inc., Alameda, CA) at 15, 30, 45, 60, 90 and 120 min post injection. Incremental area under the curve (AUC) for the blood glucose response was calculated from the baseline blood glucose concentration.

### Insulin tolerance test

Insulin tolerance tests were performed 3 h after food was removed from the rats’ cage. Blood glucose concentrations were measured as described above. After measuring the initial blood glucose concentration each rat was given an intraperitoneal injection of insulin (Humulin) at a dosage of 0.75 U/kg. Blood measurements were taken at 15, 30, 45 and 60 min post injection. Incremental area above the curve (AAC) for the blood glucose response was calculated from the baseline blood glucose concentration. Glucose and insulin tolerance tests were separated by at least 2 days.

### Metabolic caging measurements

During the last (8th) week, animals were housed individually in a Comprehensive Lab Animal Monitoring System (CLAMS, Columbus Instruments, Columbus, OH) with free access to food and water for a 24 h light-dark cycle similar to the previous 7 weeks. 24-h has been used by others as a suitable period of time to assess metabolic activity [20]. Measurements were taken during the light (8 am to 8 pm) and dark (8 pm to 8 am) phases for physical activity and oxygen uptake (VO_2_). The first 2 h of measurements were discarded to allow for acclimation.

### Insulin injection and tissue collection

Animals were fasted overnight for 6 h before being anesthetized with sodium pentobarbital (60 mg/kg body mass). From the right side of the body, red gastrocnemius muscle (RG, fast oxidative glycolytic), white gastrocnemius muscle (WG, glycolytic), subcutaneous (SQAT, inguinal) and visceral (VAT, perigonadal) adipose tissue samples were obtained and flash frozen in liquid nitrogen and stored at −80 °C for later analysis. Animals were then injected with a maximal insulin dose (7.5 U/kg). 15 min later, tissues from the left side of the animal (RG, WG, SQAT, VAT), as well as the median lobe of the liver were obtained to assess phosphorylation of AKT. Samples were flash frozen in liquid nitrogen and stored at −80 °C until subsequent analyses.

### Western blotting

*Muscle, adipose and liver samples* were homogenized in ice-cold lysis buffer for two 1-min cycles in the Fast Prep® tissue homogenizer (MP Biomedicals, Quebec, Canada) and then centrifuged at 1500 *g* for 15 min at 4 °C. The supernatant/infranatant was carefully removed and the protein concentration was determined using the BCA method; equal amounts of protein were loaded into 15 well 10 % SDS-PAGE acrylamide gels along side a colored protein ladder. The proteins were separated using approximately 1.5 h of electrophoresis at 100-130 mV and subsequently transferred onto a nitrocellulose membrane for 2 h at 200 mA (4 °C) using a Bio-Rad Mini-PROTEAN Tetra Cell system. Membranes were incubated in Tris buffered saline–0.01 % tween (TBST) supplemented with 5 % non-fat dry milk for 1 h to block non-specific protein binding. The membranes were incubated overnight at 4 °C in primary antibodies (p-AKT Thr308 and p-AKT Ser 473; Cell Signaling, Massachusetts, USA; 1:1000 dilution in TBST with 5 % BSA). Unbound primary antibody was washed away with TBST prior to incubation for 1 h at room temperature with HRP-conjugated goat anti-rabbit secondary antibody (1:2000 in TBST-1 % non-fat dry milk solution). Excess antibody was washed away with TBST followed by TBS to clear the membrane for imaging using ECL. Equal loading and transfer was determined by detecting alpha-tubulin (Sigma Aldrich, Missouri, USA).

### Statistical analysis

Statistical analysis was conducted using Prism 6.0 (GraphPad Software Inc., 2008, San Diego, USA). Two-way ANOVA was used when comparing two treatments (insulin and diet), while differences between dietary groups only were determined using a one-way ANOVA, followed by a Fisher LSD posthoc test. Statistical significance was accepted at *p* ≤ 0.05.

## Results

### Fatty acid profile of the custom butter

The content of various conjugated linoleic isomers of the non-SARA and SARA butters, as well as in the final prepared diet is shown in Table 1. This is the identical butter used in our previous study which has already been reported [13]. However, the fat content of the final prepared diet in the current study (45 % kcal) was lower than that used in our previous study (60 % kcal); therefore, the content of the CLA isomers per 100 g of diet is different than previously reported [13]. Briefly, all measured CLA isomers demonstrated some increase in the SARA butter relative to the control butter, with the total CLA content increasing by about 3-fold. In the prepared diet, the most abundant CLA isomer, c9, t11, was increased by ~2-fold (0.28 ± 0.03 vs. 0.12 ± 0.04 g/100 g diet; *p* <0.05). The t10, c12 CLA isomer was increased the most, by ~10-fold in the final prepared diet (0.024 ± 0.001 vs. 0.002 ± 0.0002 g/100 g diet; *p* <0.001).

**Table 1:** Content of linoleic acid (18:2 9c, 12c) and conjugated linoleic acid isomers in non-SARA (control) and SARA diets (g/100 g)

Fatty acid	Non-SARA diet		SARA diet	
	g/100 g of butter fat^b^	g/100 g diet^a^	g/100 g of butter fat^b^	g/100 g diet^a^
18:2 9c, 12c	1.72 ± 0.03	0.41 ± 0.007	2.50 ± 0.225*	0.60 ± 0.05*
18:2 11t, 15c	0.07 ± 0.005	0.017 ± 0.001	0.39 ± 0.002*	0.09 ± 0.005*
**18:2 9c, 11t**	**0.49 ± 0.15**	**0.12 ± 0.04**	1.16 ± 0.144*	0.28 ± 0.03*
18:2 9t, 11c	0.01 ± 0.00	0.002 ± 0.001	0.08 ± 0.004*	0.02 ± 0.001*
**18:2 10t, 12c**	**0.01 ± 0.001**	**0.002 ± 0.0002**	0.10 ± 0.003*	0.024 ± 0.001*
18:2 9t, 11t and 10t, 12t	0.03 ± 0.004	0.007 ± 0.001	0.08 ± 0.004*	0.02 ± 0.001*
18:2 11t, 13t	0.01 ± 0.002	0.002 ± 0.0005	0.03 ± 0.004*	0.007 ± 0.001*
Total CLA	0.62 ± 0.05	0.15 ± 0.012	1.84 ± 0.16*	0.44 ± 0.04*

Data presented as mean plus/minus standard deviation, in g/100 g of butter fat or prepared diet (^a^45 % kcal from butter). The main CLA isomers, 9c, 11t and 10t, 12c are bolded *, significantly different from non-SARA butter (*p* < 0.05). *n* = 4 batches of non-SARA butter, and 2 batches of SARA butter^b^ as previously reported [13]

### Body mass and food intake

At the beginning of the trial, lean Zuckers weighed 197 ± 5 g, and the fatty Zucker rats weighed 377 ± 4 g. After 8 weeks, the lean Zucker rats weighed 262 ± 10 g and the fatty Zucker rats collectively weighed 585 ± 9 g. There were no significant differences between the various fatty Zucker groups (Table 2). Average daily food intake (Table 2) during the 8 weeks was similar in all fatty Zucker groups, and significantly greater than lean Zuckers (20 ± 0.5 vs. 13.5 ± 0.3 g/d; *p* < 0.05).

**Table 2:** Mean daily food intake and final body mass

	Lean Zucker (LF diet)	Fatty Zucker (LF diet)	Fatty Zucker (Lard diet)	Fatty Zucker (SARA diet)	Fatty Zucker (non-SARA diet)
Food intake (g/day)	27.1 ± 0.6^a^	40.5 ± 0.7^b^	39.8 ± 0.8^b^	40.2 ± 0.8^b^	37.8 ± 1.4^b^
Final body mass (g)	262 ± 10^a^	546 ± 21^b^	588 ± 16^b^	593 ± 10^b^	606 ± 18^b^

Mean daily food intake over 8 weeks, and final body mass after 8 weeks. Data are presented as mean plus/minus standard error. *n* = 8−10 per group. Groups which share a letter are not statistically different. Statistical significance accepted at *p* < 0.05. *LF* low fat

### Glucose and insulin tolerance tests

**Fig. 1:**
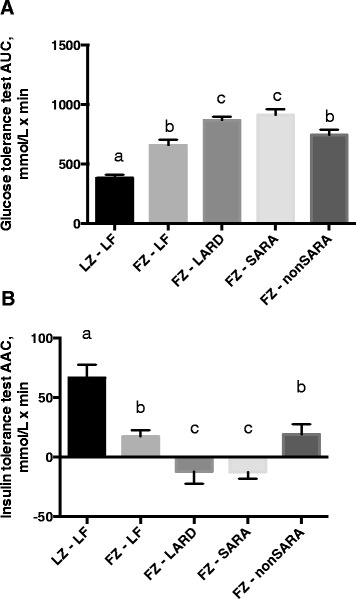
Calculated (**a**) area under the curve for glucose tolerance, and (**b**) area above the curve for insulin tolerance tests. Data are presented as mean plus standard error. *n* = 8−10 per group. Groups which share a letter are not statistically different. Statistical significance accepted at *p* < 0.05. LZ, lean Zuckers; FZ, fatty Zuckers; LF, low fat; SARA, subacute rumen acidosis

The calculated area under the curve (Fig. 1a) for glucose tolerance was greatest (*p* <0.05) in the LARD and SARA groups followed by the LF and non-SARA groups. AUC for all fatty Zucker groups was significantly greater than the lean Zucker group (*p* <0.05). The calculated area above the curve (Fig. 1b) for the insulin tolerance test showed essentially the same pattern as for the glucose tolerance test. Area above the curve was smallest (indicating poorest insulin response) in the LARD and SARA groups (*p* <0.05), followed by the LF and non-SARA groups (*p* <0.05). In fact, the LARD and SARA-butter fed fatty Zucker rats showed essentially no drop in blood glucose in response to insulin. All fatty Zucker groups demonstrated an impaired insulin response relative to the lean Zucker group (*p* <0.05).

### Physical activity and oxygen uptake

Lean Zucker rats were significantly more active during the dark (awake) phase than during the light phase (Table 3); fatty Zucker rats generally had similar activity levels during both the light and dark phases. During both the light and dark phase, lean Zuckers were significantly more active compared all fatty Zucker groups. During the dark phase, SARA and non-SARA rats were the least active groups (*p* <0.05). Only the lean Zucker rats showed a significant increase in oxygen uptake in the dark vs. the light phase. During both the light and dark phases, lean Zucker rats had a significantly greater oxygen uptake than the fatty Zucker groups (*p* <0.05).

**Table 3:** Physical activity and oxygen uptake

		Lean Zucker (LF diet)	Fatty Zuck (LF diet)	Fatty Zucker (Lard diet)	Fatty Zucker (SARA diet)	Fatty Zucker (non-SARA diet)
Activity, beam breaks	light	2902 ± 190^a^	1152 ± 189^b^	1274 ± 72^b^	855 ± 110^b^	951 ± 84^b^
	dark	4730 ± 780*^A^	1827 ± 252^B^	1572 ± 182^BC^	854 ± 70^C^	975 ± 68^C^
VO_2_, mL/hour/kg	light	21.6 ± 1.3^a^	14.3 ± 0.9^bc^	15.6 ± 0.4^b^	13.0 ± 0.7^c^	12.9 ± 0.6^c^
	dark	26.7 ± 1.5*^A^	16.2 ± 1.0^B^	16.4 ± 0.4^B^	13.3 ± 0.9^C^	13.1 ± 0.6^C^

Physical activity and oxygen uptake in Zucker rats fed various diets for 8 weeks. Data are presented as mean plus/minus standard error. *n* = 8-10 per group. Lower case letters are used to compare groups in the light phase, upper case letter are used to compare groups in the dark phase, and an asterisk denotes a significant difference between light and dark phases. Groups which share a letter are not statistically different. Statistical significance accepted at *p* < 0.05. *LF* low fat; *VO*_*2*_ oxygen uptake; *SARA* subacute rumen acidosis

### Insulin signaling

#### Muscle

**Fig. 2:**
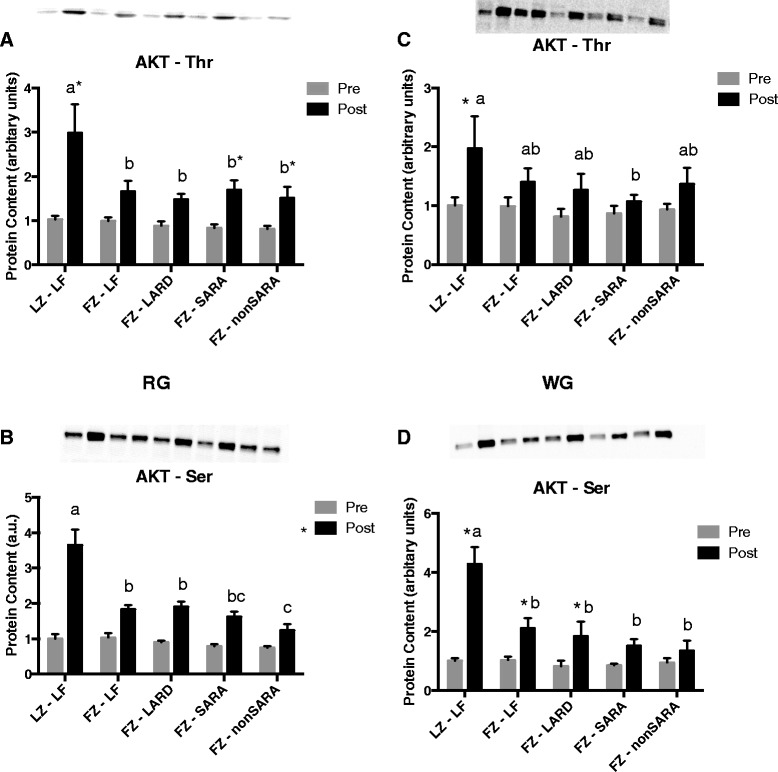
Phosphorylated Thr and Ser AKT content in red (**a**, **b**) and white (**c**, **d**) gastrocnemius muscle before and after an acute insulin injection. Data are presented as mean plus standard error. *n* = 8−10 per group. Groups which share a letter are not statistically different. * indicates a significant difference between pre and post insulin conditions. Statistical significance accepted at *p* < 0.05. LZ, lean Zuckers; FZ, fatty Zuckers; LF, low fat; SARA, subacute rumen acidosis

In both RG and WG, acute insulin injection significantly increased phosphorylation of Ser and Thr residues; this response was significantly blunted in fatty Zucker groups regardless of diet (Fig. 2). In general, there were no significant differences in AKT phosphorylation amongst the different fatty Zucker groups.

#### Adipose tissue

**Fig. 3:**
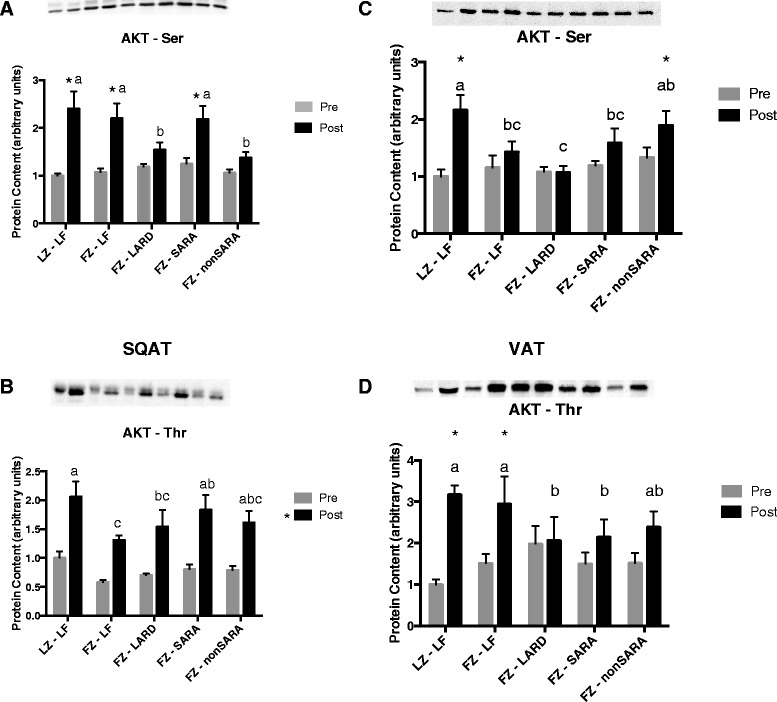
Phosphorylated Thr and Ser AKT content in subcutaneous (**a**, **b**) and visceral (**c**, **d**) adipose tissue before and after an acute insulin injection. Data are presented as mean plus standard error. *n* = 8−10 per group. Groups which share a letter are not statistically different. * indicates a significant difference between pre and post insulin conditions. Statistical significance accepted at *p* < 0.05. LZ, lean Zuckers; FZ, fatty Zuckers; LF, low fat; SARA, subacute rumen acidosis

In SQAT (Fig. 3), insulin administration significantly increased p-Ser AKT in lean Zuckers, LF and SARA fatty Zuckers, but not in the LARD and non-SARA groups (*p* < 0.05). p-Thr AKT was increased in all groups, and was the greatest in the lean Zuckers and the least in the low-fat fed fatty Zuckers (*p* < 0.05). In VAT (Fig. 3), insulin response was consistently impaired in the high fat fed fatty Zucker rats. Insulin acutely increased p-Ser AKT in lean and non-SARA Zuckers (*p* < 0.05), although the response was significantly blunted in the non-SARA group. p-Thr AKT increased in response to insulin only in the low-fat fed lean and fatty Zuckers (*p* < 0.05), but not the Lard, SARA or non-SARA groups.

#### Liver

**Fig. 4:**
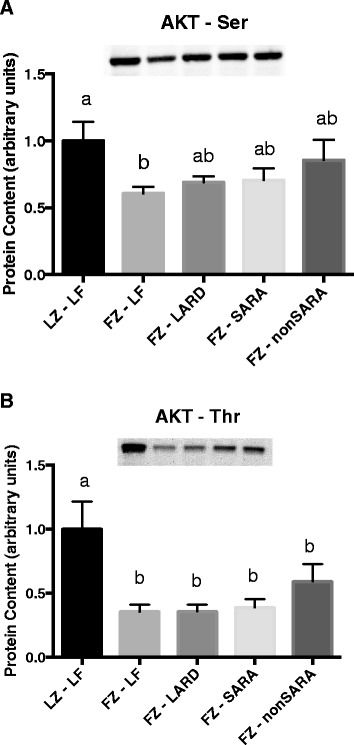
Phosphorylated Thr (**a**) and Ser (**b**) AKT content in liver after an acute insulin injection. Data are presented as mean plus standard error. *n* = 8−10 per group. Groups which share a letter are not statistically different. Statistical significance accepted at *p* < 0.05. LZ, lean Zuckers; FZ, fatty Zuckers; LF, low fat; SARA, subacute rumen acidosis

Liver AKT content was only measured post insulin injection. p-Ser AKT and p-Thr AKT content after insulin injection was generally lower in the fatty Zucker rats (Fig. 4).

## Discussion

In a recent study, we demonstrated that feeding a high fat butter-based diet enriched in CLA_t10,c12_ to lean healthy rodents caused significant, but small increases in fasting blood glucose and insulin concentrations [13]. Insulin tolerance was significantly impaired by feeding the CLA_t10,c12_-enriched butter, but we did not observe any significant impairment in either glucose tolerance or insulin stimulated muscle glucose uptake [13]. Altogether, these findings demonstrated a minimal impact of consuming large amounts of a food naturally enriched in the specific CLA isomer believed to cause insulin resistance. Therefore, the purpose of the present study was to extend these initial findings by examining the metabolic impact of feeding a CLA_t10,c12_-enriched butter to female fatty Zucker rats, which are known to develop insulin resistance and diabetes when fed a high fat diet [15]. To this end, we i) performed whole-body glucose and insulin tolerance tests, and ii) examined acute insulin signaling in response to a maximal insulin dose in skeletal muscle (red and white gastrocnemius), adipose tissue (subcutaneous and visceral depots), as well as liver. Finally, we iii) assessed changes in metabolic rate (oxygen uptake) and physical activity in a metabolic caging system.

Our current findings extend our previous observations that feeding a butter naturally enriched in CLA_t10,c12_ has a negative impact on whole body glucose homeostasis. However, unlike our previous study in which insulin, but not glucose tolerance was impaired by the CLA_t10,c12_ enriched butter [13], both glucose and insulin tolerance were impaired by the SARA (high CLA_t10,c12_) butter in the present study. The impairment of glucose and insulin tolerance was essentially identical to that induced by a diet containing similar amounts of lard. The negative impact of the diets containing lard and SARA-butter was most apparent with the insulin tolerance tests, as there was essentially no decrease in blood glucose in response to an *in vivo* insulin injection. In the current study, the amount of fat (butter, lard) provided by the diet was reduced to a more realistic content compared to our previous study (45 % kcal vs. 60 % kcal). Therefore, the reason for the more consistent negative effect of CLA_t10,c12_-enriched butter in the present study was almost certainly the choice of rodent model. We utilized fatty female Zucker rats in this study as they develop overt diabetes when placed on a moderately high fat diet i.e. 45 % kcal from fat, and are a commonly used model for human obesity. Thus, our primary hypothesis appears to be correct in that a more obesity/diabetes prone animal demonstrated a more consistent impairment in glucose homeostasis (both glucose and insulin tolerance) compared to the lean healthy rats used in our previous study in which only insulin tolerance was impaired [13].

As an extension to our previous work, we also assessed insulin signaling in two muscle fiber types (oxidative and glycolytic), two adipose tissue depots (subcutaneous and visceral), as well as the liver. In both muscle fiber types, AKT phosphorylation was consistently impaired in response to a maximal acute insulin injection in all fatty Zucker rats, regardless of their diet. Therefore, impaired maximal insulin signaling in skeletal muscle could not account for the fact that the greatest degree of glucose and insulin intolerance was observed in the LARD and SARA groups. Certainly, it is possible that insulin sensitivity rather than maximal response was affected, and that our choice of a maximal insulin dosage precluded our ability to detect any differences between groups. However, as evident in Figs. 2 and 3, the increase in AKT phosphorylation in either muscle fiber was quite small in the fatty Zucker rats, and in some cases, the increase in response to insulin was not significant. Therefore, changes in AKT phosphorylation in the Zucker rats in response to a lower submaximal concentration of insulin would likely have been undetectable. Interestingly, AKT phosphorylation in visceral adipose tissue generally appeared to be most impaired in the LARD and SARA groups. However, this was not true in subcutaneous adipose tissue in which insulin signaling did not appear to be as consistently or robustly inhibited. Finally, AKT phosphorylation in liver was generally uniformly decreased amongst the various fatty Zucker groups, with no differences between dietary groups. Overall, differences in maximally stimulated insulin signaling in any of the main insulin-responsive tissues do not account for the difference observed in our whole-body tolerance tests.

One of the limitations in our previous study was that we did not include an assessment of physical activity or oxygen consumption (metabolic rate). Therefore, in the current study, animals were placed in a metabolic caging unit to assess these parameters. As expected, all fatty Zucker groups, regardless of diet, demonstrated a significantly reduced oxygen uptake during both light and dark phases. In fact, only the lean Zucker group showed a significant increase in oxygen uptake during the dark (awake) phase. Generally, there were few differences between dietary treatments, although interestingly, both butter-fed groups (SARA and non-SARA) had a lower oxygen uptake than the LARD or low fat fed fatty Zuckers. Overall, physical activity tended to match the patterns in oxygen uptake and was greatest in lean Zucker rats regardless of light or dark phase. Physical activity was also the lowest in the butter-fed groups. Although this decrease in physical activity was not statistically significant, the percent decrease in the butter-fed groups (~25-40 % decrease relative to LARD), would explain the ~20 % decrease in oxygen uptake in these groups. As seen in Table 2, there were no significant differences in physical activity or oxygen uptake between the SARA and non-SARA groups; therefore, the impairment in glucose and insulin tolerance cannot be explained by changes in these parameters.

Collectively, our results from two studies ([13] and present) suggest that the consumption of high amounts of a food naturally enriched CLA_t10,c12_ increases the risk of developing impaired glucose and insulin tolerance, although the degree of impairment is not as great as that achieved in studies providing higher amounts of purified CLA_t10,c12_ [11]. This may be due to several reasons including i) a lower consumption of CLA in our two studies (<0.5 g CLA/100 g diet) compared to previous rodent studies using supplemental CLA (>1 g/100 g diet [4, 5]), ii) the fact that all CLA isomers, and not just CLA_t10,c12_ were altered in the SARA butter, and iii) a relatively shorter duration of feeding compared to other studies which extend up to 6 months [12]. Previous studies have demonstrated that mixed isomer CLA supplementation in obese Zucker rats can improve insulin sensitivity and inflammation [21, 22], and recently, that a CLA_c9,t11_ naturally enriched butter prevented high-fat diet induced hyperinsulinemia in rats [14]. Therefore, since the CLA_t10,c12_ isomer is the most greatly affected CLA isomer in the SARA butter, demonstrating an ~10-fold increase, it is reasonable to suggest it is this isomer that is causing the impaired glucose and insulin tolerance. However, we cannot rule out the possibility that, being a naturally altered food, other constituents may have been responsible as well.

## Conclusion

The outcome of this study indicates that the consumption of large amounts of a food naturally enriched in CLA_t10,c12_ content can significantly impair whole body glucose and insulin tolerance in a diabetes-susceptible rodent model. This outcome cannot be explained by changes in maximal insulin response in major insulin responsive tissues (skeletal muscle, liver, adipose tissue), or by changes in physical activity or energy expenditure. Although we cannot comment on whether the consumption of small of amounts of CLA_t10,c12_-enriched foods might have similar undesirable consequences, it may be prudent to examine this possibility over longer periods of time in humans.

## Abbreviations

CLA: Conjugated linoleic acid
SARA: Subacute rumen acidosis
RG: Red gastrocnemius
WG: White gastrocnemius
SQAT: Subcutaneous adipose tissue
VAT: Visceral adipose tissue
LF: Low fat
SARA: Subacute rumenal acidosis
GTT: Glucose tolerance test
ITT: Insulin tolerance test
AUC: Area under the curve
AAC: Area above the curve
LZ: Lean Zucker
FZ: Fatty Zucker
